# Fluctuations in Mediterranean Diet Adherence Pre- and Post-Pandemic: A Study of Portuguese Cohorts 2019–2024

**DOI:** 10.3390/nu16193372

**Published:** 2024-10-03

**Authors:** Vanda Lopes de Andrade, Paula Pinto

**Affiliations:** 1 Agriculture School, Polytechnic University of Santarem, 2001-904 Santarem, Portugal; vanda.andrade@esa.ipsantarem.pt; 2 Research Centre for Natural Resources, Environment and Society (CERNAS), 2001-904 Santarem, Portugal; 3 Life Quality Research Centre (CIEQV), 2040-413 Rio Maior, Portugal; 4 Research Institute for Medicines (iMed. ULisboa), Faculty of Pharmacy, Universidade de Lisboa, 1649-003 Lisboa, Portugal

**Keywords:** MEDAS, COVID-19, healthy lifestyle, sport practice, food choices, sociodemographic factors

## Abstract

Background/Objectives: The Mediterranean Diet (MD) is a lifestyle offering numerous health benefits. Nevertheless, the adherence to the MD is moderate even in Mediterranean countries. While sociodemographic factors influence MD adherence, additional impacts occurred due to the COVID-19 pandemic. This cross-sectional longitudinal study with three cohorts of Portuguese adults analyzes MD adherence before, during, and after the COVID-19 pandemic, and explores the effect of sociodemographic variables. Methods: Sociodemographic factors, lifestyle habits, and MD adherence were assessed in the years 2019, 2021, and 2024 with an online self-filled questionnaire. MD adherence was measured with the Mediterranean Diet Adherence Screener (MEDAS). Results: MEDAS score increased significantly (*p* < 0.05) from 2019 to 2021 (6.2 ± 0.7 to 7.7 ± 0.1), followed by a significant (*p* < 0.05) decrease in 2024 (7.2 ± 0.1) relative to 2019, which was more pronounced in participants with higher income. Accordingly, a trend in healthier food choices was observed followed by a decline in 2024. Of note is the significant increase in red meat consumption (*p* < 0.05) in 2024 relative to 2021. Respondents who consumed more red meat were mostly men, employed, or in a stable relationship. Most respondents practiced sport “Never or occasionally” in 2019 and 2021 (59.4 and 55.2%, respectively); in 2024, this category was significantly (*p* < 0.05) reduced (40.9%); men or higher-income participants were more likely to meet the recommended activity levels. Conclusions: This study reveals that the improvements in MD lifestyle during the pandemic were not sustained in 2024, as healthier habits formed during confinement were not fully integrated into long-term behavior. These findings strengthen the need for targeted public health interventions to promote the MD.

## 1. Introduction

The MD emerged from the traditional habits of people living in countries bordering the Mediterranean Sea. Although Portugal is bathed by the Atlantic Sea, it is considered to be a Mediterranean country due to its similarity of geography, climate, and culture with other Mediterranean countries such as Spain, Italy, and Greece [1]. Regarding food habits, the MD is characterized by a high consumption of plant-based foods, with a wide variety of vegetables, fruits, cereals, and nuts. The primary source of fat is olive oil, used both to cook and as seasoning. Protein sources include mainly poultry and fish, with a moderate intake, as well as legumes, with red meat being consumed in low amounts. Water is the main drink at meals, but red wine may be consumed in moderation. The MD is more than healthy food habits; it is a lifestyle characterized by adequate amounts of daily physical activity, mainly outdoors; adequate rest; and socialization with family and friends [2].

The MD has been linked to numerous health benefits, such as improved cognitive function and reductions in cardiometabolic diseases [3,4,5,6], cancer [7], and SARS-CoV-2 infection [8]. Due to its high fiber content, the MD also promotes a balanced gut microbiome [9]. Beyond physical health, adherence to the MD has been linked to improved mental well-being, highlighting its comprehensive benefits for overall quality of life [10,11]. Furthermore, the MD is considered to be one of the most sustainable diets, as it is a plant-based diet featuring the consumption of seasonal and locally produced food products [2].

Public health researchers and professionals are strongly motivated to identify the social and behavioral factors associated with adherence to the MD, which in turn might help tailor nutritional strategies and interventions in a more focused and efficient manner. In general, it has been suggested that diet quality follows a socioeconomic gradient in terms of education, employment, and income [12]. Previous studies also showed a significant effect of climate change awareness on sustainable and healthy eating behaviors and adherence to the MD, and that price and convenience were significant barriers in some countries [13,14]. Even so, over the last three to four decades, many countries and regions have dramatically moved into a nutrition transition stage defined by the high consumption of ultra-processed foods and significant reductions in physical activity [15]. This trend has been partially linked to globalization, which has facilitated the widespread adoption of Western-style dietary patterns [16]. Economic and related demographic changes have led to increased income, urbanization, mass media and marketing, technological advances, and global trade in services, goods, and technology [15]. Even in traditional Mediterranean countries, the adhesion to the MD is only moderate, despite numerous campaigns and governmental programs to promote healthy eating [17].

Furthermore, the world was hit by the COVID-19 pandemic, which affected healthy behaviors. The closure of restaurants during COVID-19 lockdowns led to both positive and negative changes in dietary patterns. While some people took this opportunity to cook healthier meals [18,19], others, driven by stress, leaned towards calorie-dense comfort foods such as sweetened cereals, bread, and pastries [20]. In some studies, an increase in MD adherence during COVID-19 lockdown was reported, while in other studies a decrease was noticed, with some changes associated with sociodemographic factors [21,22]. These opposite changes in MD adherence were also observed among traditional Mediterranean countries. Many studies in Spain reported an overall increase in MD adherence, with most participants falling within the high-adherence category during lockdowns despite some noting that the consumption of unhealthy foods (e.g., sugary drinks) also increased [21,22]. A concomitant increase in sedentary behavior was also observed [22]. On the other hand, most of the studies in Italy have shown a low percentage of participants with a high adherence to the MD [22]. An overall trend in MD adherence could not be deduced from the Italian studies; some reported an increased adherence to the MD during lockdowns, particularly homemade foods, in male participants with an older age and higher income; other reported decreased adherence, with increased consumption of processed and sugary foods [22]. In Cyprus, no significant changes in MD adherence were observed before and during lockdown [21], although Cypriots residing in rural areas who were unmarried, male, and physically active were more likely to be adherent to the MD, and mild increases in MD adherence during lockdown were reported, while the Croatian population experienced a decrease in MD adherence during lockdown [22]. In Greece, elevated MD compliance was more frequently noted in females, individuals with a better family annual income, and those living with others during the COVID lockdown [23].

The World Health Organization (WHO) recognizes the importance of sport events as a channel for reaching many people and the influence of sport events at all levels of society, as well as their potential to impact human behavior, well-being, and physical and mental health [24]. However, during the COVID-19 pandemic, there were also changes in physical activity-related behaviors. Working from home and the closure of gyms further exacerbated sedentary behaviors, as commuting time was eliminated and prolonged periods were spent sitting in front of screens [25]. The COVID-19 pandemic had a significant impact on physical activity levels, with several studies suggesting that almost half of the participants became less active during the quarantine, which in turn was associated with lower subjective well-being and lower health-related quality of life [26]. Another study showed that perceived neighborhood safety, education, and pet ownership were associated with meeting physical activity guidelines during the early months of the COVID-19 pandemic, but associations differed by income [27]. In addition, research across the world estimated the prevalence of sleep disturbances to be between 21.9 and 55.8%, which in turn were significantly associated with anxiety and depressive symptoms during this period [26]. Concomitant increases in common mental health difficulties from the start of the pandemic occurred, with anxiety and depression disproportionately affecting particular groups, such as younger individuals, non-White ethnicity, economically disadvantaged persons, and females [28].

A lot of scientific evidence has been published on the effect of the COVID-19 pandemic on MD adherence and lifestyle changes, but the information is very scarce in the periods after the end of the pandemic. In the present paper, we propose to analyze eventual changes in adherence to the MD in three Portuguese adult cohorts, covering the periods of pre-confinement due to the COVID-19 pandemic (2019), two months after the last lockdown (2021), and about one year after the official end of the pandemic (2024). When studying Mediterranean dietary adherence and lifestyle behavior, it is crucial to consider sociodemographic parameters such as education level, gender, and income because these factors significantly influence dietary choices and adherence to specific diets [23,29,30]. Thus, we propose to explore sociodemographic factors that may be associated with changes in MD adherence in the studied cohorts. We aimed to determine if the adherence to the MD varied significantly among different sociodemographic groups during various phases of the pandemic and assess whether these changes were permanent or temporary. Our work hypothesis is that variations occurred and that the trend of these variations were kept over time.

## 2. Materials and Methods

### 2.1. Study Design and Ethics

This is a longitudinal cross-sectional study of three cohorts in Portugal (2019, 2021, and 2024) with the aim of exploring changes in MD adherence in Portuguese adults. This study was approved by the Ethics Committee of the Research Unit of the Polytechnic Institute of Santarém (Document 022019Agrária) and meets the terms of the European Regulation on Data Protection [31]. This study implied the application of a food frequency questionnaire designed in 2019 within a research consortium and validated for the calculation of the 14-MEDAS (Mediterranean Diet Adherence Screener) score [32]. The questionnaire was constructed on Google Forms (free version) and disseminated through institutional mailing lists, social media, and the personal contacts of the researchers involved in the study in three periods: 29 April 2019 to 28 January 2021 (the questionnaire opened after the outbreak of the COVID-19 pandemic and closed about one and a half months before the start of the first lockdown); 27 May 2021 to 27 November 2021 (the questionnaire opened two months after the end of the second lockdown); and 19 February 2024 to 3 April 2024 (about 9 months after the official statement of the end of the COVID-19 pandemic). The questionnaire was confidential, filled anonymously, and included persons with Portuguese nationality with an age ≥ 18 years and living in Portugal.

### 2.2. Definition of Variables

The complete explanation of the questionnaire has been presented in a previous paper [11]. The present study includes an analysis of the following variables from the questionnaire: (i) sociodemographic data—gender, age, marital status, education level, employment status, and household income; (ii) lifestyle—sports practice (meaning sport or intense physical activity, but for simplification purposes will be referred to as sport), sleep habits, time spent in nature, with family, or with friends, and daily meal companionship; and (iii) MD adherence—the frequency of consumption of 14 food items used to calculate the validated 14-MEDAS score (presented in the table in Section 3.2).

For posterior data treatment, the following classes were defined for sociodemographic data: gender, “Men” or “Women”; age, “Less than 45 years” or “45 years or more”; marital status, “Married or analogous relationship” or “Single, divorced, separated or widowed”; education level, “Without university level” or “With university level”; employment status, “Employed” or “Student, unemployed, housework or pensioner”; and monthly net income, “<2× Portuguese Social Support Index (PSSI)” or “>2× PSSI”, with the PSSI being EUR 438.81 in 2019 to 2021 and EUR 509.26 in 2024.

For the lifestyle and MD adherence variables, a score was attributed to the different frequencies: lifestyle—sports practice, “Never or occasionally” = 1, “Regularly, less than 150 min per week” = 2, and “Regularly, more than 150 min per week” = 3; time spent in nature, with family, or with friends, “Never or occasionally” = 1, “Sometimes” = 2, and “Frequently or almost all the time” = 3; daily meal companionship, “Alone” = 0 and “In the company of colleagues, friends or family” = 1; MD adherence—portions of red meat and derivate per week, “One or less” = 0, “Two or four = 1; “Five to six” = 2, and “Seven or more = 3”; sweets per week, “Less than one” = 0, “One” = 1; “Two” = 2; ”Three = 3”, and “Four or more” = 4; portions of nuts and fish per week and portions of vegetables and fruit per day, “Less than one” = 0; “One” = 1; “Two” = 2, and “Three or more” = 3; soup spoons of olive oil per day, “One or less” = 0, “Two or three” = 1, and “Four or more” = 2; sweet beverages per day, “Less than one” = 0, “One” = 1, and “More than one” = 2; and wine, “Occasionally” = 0, “Sometimes but not daily” = 1, “One or two glasses per day” = 2, and “More than two glasses per day” = 3. Based on these criteria, a mean score was calculated for each variable. The MEDAS score was calculated as reported previously [11] (0 = minimum, 14 = maximum). The categories of adherence to the MD were defined as weak adherence (14-MEDAS ≤ 5), moderate-to-fair adherence (14-MEDAS = 6–9), and good or very good adherence (14-MEDAS ≥ 10) [33].

### 2.3. Data Analysis

Statistical analysis was performed with the Statistical Package for the Social Sciences (SPSS) 26 statistical package for Windows (SPSS, Inc., Chicago, IL, USA). The presence of nominal and ordinal variables as well as testing scale variables for normality and heteroscedasticity, measured by the Kolmogorov–Smirnoff and Levene tests, rendered the use of non-parametric analysis as the best choice [34].

Sociodemographic variables, lifestyle variables, and the categories of adherence to the MD and individual food items are expressed as frequencies (%); the 14-MEDAS score is expressed as mean and standard deviation (sd). To assess differences in sociodemographic variables among the cohorts of the years 2019, 2021, and 2024, Mann–Whitney U tests were used for the ordinal and scale variables, while Chi square tests were used for the nominal variables. Since sex, age class, and marital status exhibited significant differences among the three cohorts, all posterior analyses were performed considering these variables as confounders. Therefore, the differences in the lifestyle and MD variables among the cohorts of 2019, 2021, and 2024 were accessed using Quade non-parametric ANCOVA tests, fixing the already mentioned sociodemographic confounders—sex, age, and marital status—as covariates. For all of the tests, differences were considered significant when *p*-values < 0.05.

Significantly different lifestyle and MD variables among the cohorts of 2019, 2021, and/or 2024 were posteriorly scrutinized to identify the sociodemographic profiles of participants in need of intervention, or participants who exhibited improvements in their lifestyle and MD adherence. Specifically, for lifestyle variables that exhibited a significant (*p* < 0.05) increase in the mean frequency score from 2019 to 2021, from 2021 to 2024, and/or from 2019 to 2024, the sociodemographic profile of participants falling in the class with the highest frequency score was characterized. For the variables with significant (*p* < 0.05) reductions in the mean frequency score from 2019 to 2021, from 2021 to 2024, and/or from 2019 to 2024, the sociodemographic profile of participants falling in the class with the lowest score was characterized. The adherence to the MD questions followed a similar methodology, using the upper or lower recommended frequency of each food item as the threshold (presented in the table in Section 3.2). In other words, when there was a variation according to the desirable trend (towards recommendations), the sociodemographic profile of participants with a score on the food item within the recommendation was characterized; when there was a variation according to an undesirable trend (away from recommendations), the sociodemographic profile of persons with a score on the food item not following the criteria was characterized. To eliminate the influence of sociodemographic differences in the global sample, the ratios were calculated with relative %s. For example, the ratio of men/women for lifestyle variables = [(Number of men in the highest or lowest frequency score/number of men in the cohort)/(Number of women in the highest or lowest frequency score/number of woman in the cohort)]. Accordingly, the ratio of men/women for MD variables = [(Number of men in the upper or lowest recommended frequency/number of men in the cohort)/(Number of women in the upper or lowest recommended frequency /number of women in the cohort)]. Only ratios equal to or higher than 2 are presented, and the complete results are presented in the Supplementary Materials (Figures S1a–d and S2a–j).

## 3. Results

### 3.1. Sociodemographic and Lifestyle Characteristics

In this longitudinal cross-sectional study, a validated questionnaire to assess MD adherence was distributed and filled out online in three periods: 2019 (506 responses), 2021 (382 responses), and 2024 (224 responses). After the exclusion of responses that did not give authorization to use the data, Portuguese participants not living in Portugal, participants without Portuguese nationality, and participants aged below 18 years, the final cohorts for analysis consisted of 500 participants in 2019, 375 in 2021, and 207 in 2024. The three cohorts included mostly women, comprising 71.8, 58.1, and 71.5% of the cohorts in 2019, 2021, and 2024, respectively (Table 1). In 2019 and 2021, most of the respondents were less than 45 years old (69.3 and 63.5%, respectively), and in 2024 the % of participants within this age category was 44.0% (Table 1). The proportions of people “married or in an analogous relationship” vs. “single, divorced, separated or widowed” were 45.0, 55.2, and 57.0% in the years 2019, 2021, and 2024, respectively (Table 1). More than 70% of participants in all three subsamples had a university-level education and were employed (Table 1). The great majority of participants had an income more than twice the Portuguese Social Support Index (PSSI): 88.0% in 2019 and 2021, and 85.5% in 2024 (Table 1). Significant differences (*p* < 0.05) among the years of 2019, 2021, and 2024 were found for the following sociodemographic parameters: gender (*p* < 0.001), age (*p* < 0.001), and marital status (*p* = 0.002) (Table 1); therefore, these parameters were treated as confounders in the subsequent analysis.

Regarding lifestyle characteristics, most of the respondents of both the 2019 and 2021 cohorts were sedentary, with 59.4 and 55.2% reporting sport practice “never or occasionally”, respectively. In 2024, the % of persons included in this category was lower (40.9%), being significantly different from 2019 (*p* = 0.001) and 2021 (*p* = 0.012) (Table 2). No significant differences among the three years were observed in sleeping habits (*p* > 0.005), with most participants reporting sleeping less than seven hours per night (Table 2). Regarding “time spent in nature”, a similar % among categories was observed in 2019 and 2021. However, in 2024, a decrease was observed in “time spent in nature”, with 43.0% of persons answering “never or occasionally” and 26.1% responding “frequently or almost all the time”, with the difference between the years of 2019 and 2024 being significant (*p* = 0.030) (Table 2). Most of the respondents spent time with family “frequently or almost all the time” (Table 2). However, a significant difference was observed in “time spent with friends” among the three years (*p* < 0.05), with 24.8%, 54.7%, and 38.2% of participants reporting “never or occasionally” in 2019, 2021, and 2024, respectively (Table 2). Most of the respondents had their meals in the company of colleagues, friends, or family, with percentages of 82.6, 76.8, and 81.6% in 2019, 2021, and 2024, respectively. Despite still being in the majority, the decrease observed in 2021 was significant (*p* =0.005) (Table 2).

### 3.2. Adherence to the Mediterranean Diet

The adherence to the MD, accessed by the 14-MEDAS score, shows variations among the years of 2019, 2021, and 2024. There was an increase from 2019 to 2021 (6.2 ± 0.7 to 7.7 ± 0.1), followed by a decrease from 2021 to 2024 (7.2 ± 0.1 in 2024) (Table 3). When considering the categories of weak, moderate-to-fair, and good-to-very good adherence, significant differences were found among the three years with an increasing trend seen from 2019 to 2024 in the classification “moderate to fair” (Table 3).

An analysis of the frequency of consumption of the food items used to calculate the MEDAS score is presented in Table 4. More than 95% of the participants reported using olive oil as their main source of fat in all three cohorts, and most consumed “two or three” soup spoons of olive oil per day (53.8, 58.9, and 49.8% in 2019, 2021, and 2024, respectively) (Table 3). A significant decrease (*p* = 0.027) was found regarding the amount of olive oil consumed between 2021 and 2024 (Table 4).

The plant-based foods included in the questionnaire were vegetables, legumes, fruits, and nuts. The highest percentage of participants consumed two portions of vegetables per day, although significant variations were observed across the years (*p* < 0.005). In 2019, 38.4% of participants consumed two portions per day, increasing to 45.6% in 2021, and then decreasing to 28.5% in 2024 (Table 3). No trend in legume intake frequency was observed across the years (Table 3). Regarding fruit consumption, in 2019, 39.0% of participants consumed two portions per day, which decreased to 34.9% in 2021, and then increased to 40.6% in 2024. The observed differences in fruit consumption between 2021 and the other two years (2019 and 2024) were significant (*p* < 0.05) (Table 3). A low intake of nuts was observed in all samples, with the highest percentage of participants consuming less than one portion per week: 34.6% in 2019, 31.7% in 2021, and 32.9% in 2024; a global trend of diminished consumption in 2024 explains the significant differences found relative to 2021 and 2019 (*p* < 0.05) (Table 4).

Considering animal protein sources, alternative sources to red meat was the main choice of participants: 76.6% in 2019, 77.1% in 2021, and 78.3% in 2024 (Table 3). Nevertheless, most participants consumed two to four portions of red meat and derivates per week, with similar percentages in 2019 (44.0%) and 2024 (44.9%), and a lower percentage in 2021 (41.1%). A significant difference was noted between 2021 and 2024 (*p* = 0.008) (Table 3). Regarding fish intake, an increase in fish consumption was noted in 2021, with 38.7% of participants consuming two portions per week and 39.7% consuming three or more portions per week, compared to 2019 (35.8% for two portions and 34.6% for three or more) and 2024 (36.2% for two portions and 31.4% for three or more). The observed differences between 2021 and the other two years (2019 and 2024) were significant (Table 4).

The intake of fats such as butter, margarine, or cream was low in all the samples, with most participants reporting less than one portion per day, with similar percentages in 2019 (68.0%), 2021 (70.1%), and 2024 (63.8%) (Table 3). The reported intake of sugar was also low. The most frequently chosen number of sweet/fizzy beverages consumed was “less than one per day”, with 82.6, 91.2, and 84.5% of participants selecting this option in 2019, 2021, and 2024, respectively; of note is the significant decrease in the consumption of these types of drinks in 2021 compared to 2019 and 2024 (*p* < 0.005) (Table 3). In terms of sweet consumption, the lower intake categories (“Less than one” and “One”) predominated across the years. A significant difference was observed between 2024 and 2021 (*p* = 0.009), although no clear trend was identified (Table 4).

Regarding wine, most participants consumed wine only occasionally, with 75.6, 77.9, and 75.8% of participants selecting this option in 2019, 2021, and 2024, respectively. A significant difference was observed between 2021 and 2019 (*p* = 0.017) (Table 4).

Finally, for dishes cooked with tomatoes or tomato sauce, onion and (or) garlic, and olive oil per week, most participants reported consuming such dishes two or more times per week: 67.6% in 2019, 67.2% in 2021, and 66.7% in 2024 (Table 4).

### 3.3. Sociodemographic Profiles of the Studied Cohorts for Lifestyle and Mediterranean Diet Adherence Parameters Which Were Significantly Different among the Years 2019, 2021, and/or 2024

**Figure 1:**
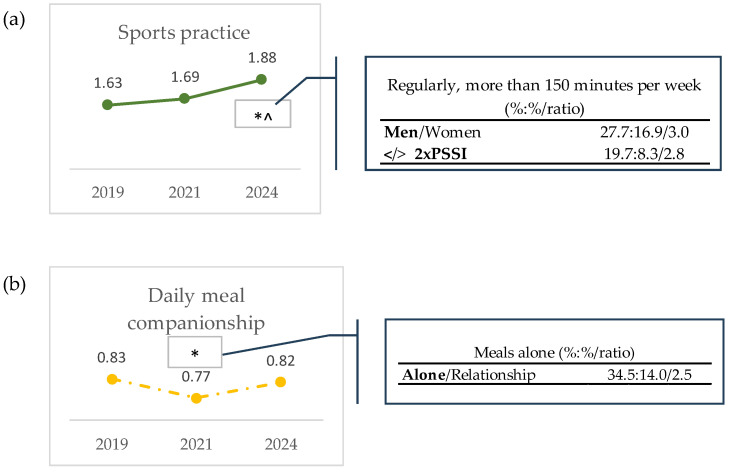
Trends in lifestyle parameters for which significant differences (*p* < 0.05) were observed and sociodemographic profiles of participants. Lifestyle parameters are expressed as means of frequency values: (**a**) sport practice: “Never or occasionally” = 1, “Regularly, less than 150 min per week” = 2, and “Regularly, more than 150 min per week” = 3; (**b**) daily meal companionship, “Alone” = 0 and “In the company of colleagues, friends or family” = 1. To access differences among cohorts, Quade non-parametric ANCOVA tests were used, fixing confounders (the sociodemographic variables sex, age and marital status) as covariates. Differences were considered significant when *p*-values < 0.05: * is different from 2019 and ^ is different from 2021. The tables show the ratios of the relative %s of sociodemographic classes of the participants in the upper-frequency category for the variables that increased (sport practice “Regularly, more than 150 min per week”), and of the participants in the lower-frequency category for the variables that decreased (meal companionship, “Alone”). Only ratios equal to or higher than 2 are presented, with the complete results presented in Supplementary Materials (Figure S1a–d).

For those lifestyle and DM variables that showed a significant (*p* < 0.05) increase or reduction in any of the studied cohorts, the sociodemographic profiles of participants who exhibited improvements in their lifestyle and met MD recommendations, or who showed a decline, was explored by calculating the ratio between the relative %s of the sociodemographic classes. Regarding lifestyle variables, only sport practice and daily meal companionship showed ratio values superior to 2-fold for gender (men/women), income (>/< 2× PSSI) (sport practice, Figure 1a), and marital status (alone/in a relationship) for daily meal companionship (Figure 1b). The mean frequency score of the variable “Sports practice” increased in 2024 relative to 2019 and 2021. The participants included in the upper category of this indicator, “Regularly, more than 150 min per week” in 2024 were mainly men (3-fold more than women) and persons earning twice or more than the PSSI (2.8-fold more than persons with lower income) (Figure 1a). Regarding conviviality at meals, in 2021 there was a significant (*p* < 0.05) decrease in the mean frequency score of the variable “Meal companionship” when compared with 2019 (Figure 1b). In fact, 2.5-fold more participants that were single, divorced, separated, or widowed had their meals alone, compared to the ones who were married or were in an analogous relationship (Figure 1b). No or very small ratios (1.0- to 1.5-fold) were observed for the other sociodemographic classes (age more/less than 45 years, with/without university education, employed/non-employed; Supplementary Materials Figure S1). The other two lifestyle variables that showed significant variations among the cohorts (time spent in nature, and time spent with friends; *p* < 0.05) showed no or very small ratios (1.0- to 1.5-fold) in all sociodemographic classes (Supplementary Materials Figure S1).

**Figure 2:**
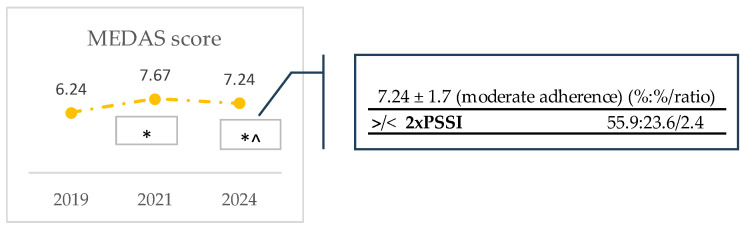
Trends in MEDAS score and sociodemographic profile of participants. To access differences among cohorts, Quade non-parametric ANCOVA tests were used, fixing confounders (the sociodemographic variables sex, age, and marital status) as covariates. Differences were considered significant when *p*-values < 0.05; * is different from 2019 and ^ is different from 2021. The tables show the ratio of relative %s of the sociodemographic classes of those participants who scored a MEDAS above the mean score of the cohort. Only ratios equal or higher than 2 are presented. All results are shown in Supplementary Materials (Figure S2a).

Regarding adherence to the MD, the sociodemographic profiles for the 14-MEDAS score and each food for which significant differences were found among cohorts were explored. No specific sociodemographic profile could be found for the observed increase in the mean MEDAS score from 2019 to 2021, as sociodemographic variables showed small differences in the range of 1.0 to 1.3 (Supplementary Materials, Figure S2a). On the other hand, the observed decrease in 2024 was more pronounced in participants with higher income. This is patent on the observation that the percentage of participants earning more than twice the PSSI who had an adherence of 7.24 ± 1.7 (moderate-to-fair adherence) was 2.4-fold greater than the persons with lower incomes and the same mean adherence (Figure 2).

**Figure 3:**
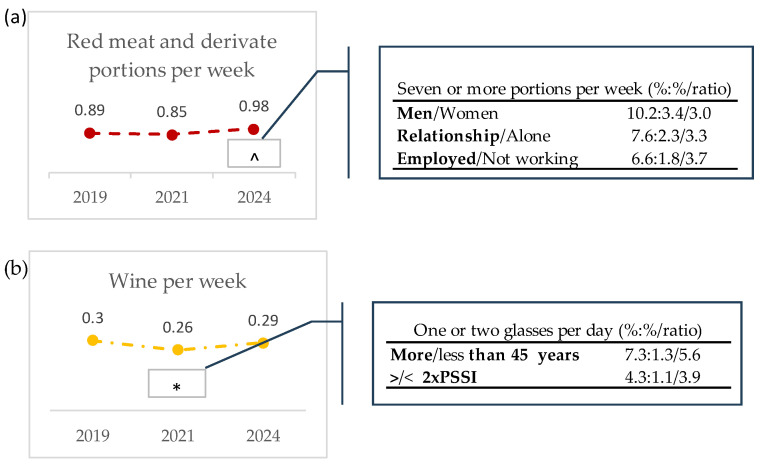
Trends in the consumption of the food items of the MD for which significant differences (*p* < 0.05) were seen among the 2019, 2021, and 2024 cohorts, and the sociodemographic profiles of participants. Frequencies of consumption are expressed as mean score values; (**a**) red meat and derivatives per week: “One or less” = 0, “Two or four = 1; “Five to six” = 2, and “Seven or more = 3”portions; (**b**) wine per week: “Occasionally” = 0, “Sometimes but not daily” = 1, “One or two glasses per day” = 2, and “More than two glasses per day” = 3. To access differences among cohorts, Quade non-parametric ANCOVA tests were used, fixing confounders (the sociodemographic variables sex, age, and marital status) as covariates. Differences were considered significant when *p*-values < 0.05; * is different from 2019 and ^ is different from 2021. The tables show the sociodemographic characteristics of the participants falling within the MD recommendations, in case of a trend towards the recommendation (wine), or participants not meeting the MD recommendations, in case of a trend away from the recommendation (red meat). Only ratios equal to or higher than 2 are presented. All results are shown in Supplementary Materials (Figure S2b–j).

When analyzing individual foods, no sociodemographic profiles could be assigned to the significant differences for vegetables, fruit, nuts, fish, sweet drinks, and desserts, as all the sociodemographic parameters showed low differences, ranging from 1.0- to 1.7-fold (Supplementary Figure S2b–j). However, a different pattern was observed for meat and wine consumption. As shown in Figure 3, the respondents increased red meat consumption in 2024 when compared with 2021. Considering that the MD recommendation is to consume less than one portion of red meat per day, when looking at the respondents that did not meet this recommendation (consumers of seven or more portions per week), it was observed that the percentages of employed people, persons in a stable relationship, or male respondents that consumed more that the recommended portions of red meat were 3.7-, 3.3-, and 3.0-fold higher than non-employed, single, divorced, separated or widowed, and female participants, respectively (Figure 3a). Wine consumption per week decreased significantly in 2021, as compared with 2019. The percentage of participants in the 2021 cohort that met the recommendation of consuming one or two glasses per day was 5.6-fold higher in participants older than 45 years, compared to participants younger than 45 years, and was 3.9-fold higher in participants with higher income (twice the PSSI) than those with a lower income (Figure 3b).

## 4. Discussion

### 4.1. MD Adherence

This paper presents a cross-sectional longitudinal study with three cohorts of Portuguese adults covering periods before the first lockdown of the COVID-19 pandemic (2019), after the last lockdown (2021), and after the end of the pandemic (2024). The aim of this study was to explore any changes in MD adherence, food consumption patterns, and other MD-related lifestyle characteristics occurring during these periods.

The mean MEDAS score showed an increase from 2019 to 2021 (6.2 ± 0.7 to 7.7 ± 0.1), followed by a decrease in 2024 (7.2 ± 0.1 in 2024). Even so, the 2024 mean value was significantly higher than the 2019 mean. This trend of an increase in MD adherence from 2019 to 2024 may be explained by the consistent increase in the percentage of participants in the moderate-to-fair adherence category (67.4, 68.8, and 75.5% in 2019, 2021, and 2024, respectively), along with a decrease in the weak adherence category relative to 2019 (31.6, 13.1, and 15.9, in 2019, 2021, and 2024, respectively). Despite the increase observed in the good or very good adherence category in 2021, only less than a quarter of the participants fell in this category. These results are in line with a study undertaken in Portugal in 2020, with data collected during the COVID-19 pandemic, two months after the first lockdown (1000 participants), which showed that only 26% of the participants fell in the category of good or very good adherence to the MD [35].

The rise in MD adherence observed in our study has also been reported in several European countries. A multi-center, cross-sectional study with 16 European countries, including Portugal, showed an increase in MD adherence across all countries during the COVID-19 lockdown period (data collected between 20 March 2020, before the first lockdown, and 5 May 2020, during the first lockdown in Portugal). MD adherence was higher in the Southern Mediterranean, with Portugal reaching the highest MEDAS scores, 6.37 and 7.34, before and during lockdown, respectively [36]. In our study, the pre-lockdown MEDAS score is similar (6.2) and the MEDAS score after the second lockdown in 2021 is higher (7.7) than that reported during the first lockdown by Molina-Montes et al. [36], suggesting that MD adherence continued to rise during the whole period of the pandemic.

In fact, it was our initial belief that there could be an increase during the confinement could be due to more time being spent at home and the opportunity to cook meals. Moreover, during the COVID-19 lockdown, Portugal launched several initiatives to promote healthy diets, with a focus on the MD. The Directorate-General of Health’s National Healthy Eating Promotion Program (PNPAS) published the Mediterranean Diet Wheel as a food guide to increase adherence to this eating pattern, along with the national expansion of the database of local and seasonal products, producers, markets, and baskets [37]. Additionally, the project “O prato certo” (The Right Plate) became part of a collaborative platform providing practical information on the MD, such as seasonal food tips, recipes, and best practices [38].

On the other hand, a recent narrative review reported that a substantial number of studies across European countries found no changes in MD adherence during the COVID-19 lockdown period [22]. According to this review, individuals receiving some form of lifestyle intervention, or who had more time at home to plan and cook meals, had better adherence to the MD, while their unassisted counterparts, or people with higher stress, anxiety, screen time, or boredom had lower adherence.

Furthermore, our results show a drop in MEDAS score from the end of the second lockdown to about one year after the end of the pandemic (7.7 to 7.2, respectively), suggesting that the behavior changes observed during the pandemics could not hold. Though the consumption of healthy food has previously been related to higher income in Portuguese adults [39], our study found that the decrease in MEDAS score in 2024 was more pronounced in participants with higher income. Several studies exist on changes in eating habits and lifestyle due to the COVID-19 pandemic during or immediately after the strict lockdown period, but very few were performed about their long-term impacts. Even so, a study of a cohort of Italian adolescents showed an increase in the daily consumption of sweets, fried foods, and UPFs during the lockdown period. Over a 2-year period, the percentage of adolescents who reported consuming UPFs daily increased, with a greater relative increase in the rate of adolescents consuming these foods more than thrice a day (from 16.2% to 22.7%). Such was one of the most worrying long-term consequences of the change in food habits: the excessive consumption of UPFs [40]. However, it should be considered that in our study, an opposite trend occurred during the lockdown and that the sampled population are adults.

One reason for the long-term decrease in MEDAS score might be the escalation of the Russia–Ukraine war in 2022, which led to rising prices for food and energy. Moreover, although global food and fuel prices have receded from their peak levels at the inception of the conflict in February 2022, they remain high compared with pre-conflict levels. Notably, as of December 2023, food and grain prices were still roughly 12–13% above their December 2020 levels. The lack of availability of food, and instability around its supply, turned more people “food-insecure” [41,42]. In Portugal, the price of food has almost doubled since the invasion of Ukraine. Olive oil, rice, and hake are some of the products that have become more expensive, with such foods being included in the MD [43].

A consumer survey on 10 European countries has shown that 81% of the participants changed their food choices, with increased price sensitivity and mindful eating being the most common reported changes [30].

When analyzing the intake frequency of the individual food items used to calculate the MEDAS score, we observed a trend in healthier food choices in foods such as vegetables, fish, red meat, and sweet beverages during the COVID pandemic, followed by a decline in 2024. The percentage of participants that met the MD recommendation of two or more vegetable servings per day was 53.6% in 2019, peaking at 2021 (62.7%) and followed by a significantly (*p* < 0.05) sharp decline in 2024 (40.6%), which was not related to any sociodemographic profile of our participants. The patterns in fruit consumption revealed a significant decrease in 2021 compared to 2019 and 2024, with percentages dropping from 39.0% in 2019 to 34.9% in 2021 before rising to 40.6% in 2024. Similarly to vegetable consumption, the observed differences in fruit intake could not be related to any sociodemographic profile. Another Portuguese study undertaken during the COVID pandemic reported that only 39% of Portuguese adults met the fruit serving recommendation and 48% met the vegetable serving recommendations of the MD [35].

Portugal is the leading country in Europe in fish consumption, with 59.9 kg consumed per inhabitant in 2019 (quantity in live weight) [44], which corresponds to about 1.2 kg per week. Accordingly, we observed a high percentage of participants consuming two and three or more portions per week, with a rise from 70.4% in 2019 to 78.4% in 2021 and then a significant decline to 67.6% in 2024. Regarding red meat consumption, our study showed a consistent preference for consuming two to four portions of red meat per week, with a slight decrease from 2019 to 2021 (44.0% to 41.1%, respectively) and then an increase in 2024 to 44.9%, particularly in men, participants in a stable relationship, and employed participants. Several other studies have shown evidence that men eat more red meat than woman [29,45,46,47], as do employed people [46].

The Global Burden of Disease Report shows that in 2021, around 210,000 years of healthy lives were lost due to dietary risks in Portugal. The main dietary risks were the low intake of whole grains (29.6%) and fruit (12%), and high intake of red meat (21%) and processed meat (15%) [48]. Our results highlight the need for public health interventions with gender-specific campaigns to decrease the consumption of red meat and increase the consumption of vegetables and fruits.

Of note was the high percentage of participants consuming less than one sweet beverage per day, peaking at 91.2% in 2021, and then suffering a rebound in 2024 to 84.5%, reinforcing the idea that any healthy choices maintained during the COVID-19 pandemic did not hold long after the end of the pandemic.

### 4.2. Lifestyle

The characteristics of the Mediterranean Diet include not only its dietary pattern (“what is eaten”) but also the cultural and social activities related to food and various lifestyle aspects, including the regular practice of physical activity and conviviality [49]. In this study, both aspects exhibited changes from 2019 to 2024.

A global trend for an increase in sport practice was observed from 2019 to 2024 (Table 2 and Figure 1a). However, it should be mentioned that despite the known health and well-being benefits of sport practice, the pre-pandemic levels of physical activity in the European Union (EU) were worryingly low. In 2015, a European study reported that only 61.5% of European adults attained the physical activity recommendations of the World Health Organization (WHO) criteria—to engage in at least 150 min/week of moderate-to-vigorous physical activity [50]. In 2019, only 44.3% of the European population practiced physical activity at least once a week and in Portugal these numbers dropped to 33.2% [51]. Our results are in accordance with these data, as most of the respondents answered that they practiced sports “Never or occasionally” (59.4, 55.2, and 40.9% in 2019, 2021, and 2024, respectively). While more than 50% of the participants reported sedentary behavior in 2019 and 2021, a significant decrease was observed in 2024 (Table 2). This may be in line with the fact that before the COVID-19 pandemic, sports activities slightly increased in the EU between 2014 and 2019 [51]; thus, this trend may have been recovered after the end of the pandemic.

In March 2020, the WHO declared the COVID-19 outbreak to be a global pandemic, and until 19 January 2022, over 325,000,000 confirmed cases were diagnosed in more than 130 countries and areas, resulting in approximately 5,500,000 deaths [52]. Due to lockdown, the entire sports world was impacted, most particularly non-professional sports clubs and federations, which, owing to their non-profit status, faced liquidity shortages and even bankruptcy due to the lack of revenue [53]. Sedentary leisure behaviors increased, while time spent in physical activity (absolute time and intensity adjusted) declined [39]. At a first glance, it would be expected that our study revealed a global decrease in sport activity from 2019 to 2021; however, a trend of a slight increase was denoted, although not significant (*p* > 0.05), Figure 1a. Quite interestingly, the % of respondents who practiced sports “Never or occasionally” diminished from 59.4% to 55.2% between the years of 2019 and 2021, and as such was concomitant with an inverse trend as regards persons who practice sports either less or even more than 150 min per week (the category “Regularly, less than 150 min per week” increased from 18.2% in 2019 to 20.7% in 2021, and the category “Regularly, more than 150 min per week” increased from 22.4% in 2019 to 24.1% in 2021) (Table 2). An explanation for these results might be that exercising at home in a limited space and without any equipment was still possible during lockdown. In fact, great efforts were undertaken at several levels by different societal actors to maintain the population’s physical activity during this period. Fitbit data, a popular exercise tracker, reveals that a massive rise in non-gym exercises occurred during the pandemic. Data from Strava, which is a social network where around 70 million people globally record and share exercise sessions, reveal that the number of people setting records for cycling or running a particular stretch was up 50% between April and June 2020 [53]. Additionally, many fitness studios reduced rate subscriptions to apps and online classes; live fitness demonstrations were also available on social media platforms [53,54,55]. Furthermore, individual outdoor sports gained growing popularity in Europe. A survey in France, Germany, Italy, Poland, Spain, Sweden, and the UK showed that 70% of respondents were looking to participate in outdoor activities after the lockdown [53].

New perspectives and consequent habits acquired from the pandemic by the general population possibly contributed to the significant (*p* < 0.05) increase in sport practice observed in 2024, relative to both 2021 and 2019 (Table 2 and Figure 1a). There have been extensive efforts in the Western world to raise public awareness about mental health problems, with the goal of reducing or preventing these symptoms across the population [56]. In Canada, while some unhealthful behaviors appeared to have been exacerbated, other more healthful behaviors have emerged since COVID-19 [57]. It is a possibility that the pandemic fostered an increase in the population’s awareness of mental health and well-being and its connection with sports, and that such a trend is still ongoing nowadays; further studies are necessary to consubstantiate such a hypothesis.

The proportion of men who performed physical activity “Regularly, more than 150 min per week” was 3-fold higher than women (Figure 1a). A survey performed in 2019 among the 24 EU countries showed that more men (47%) than women (42%) exercised regularly [51].

Data from this study additionally show that the respondents included in the category of practicing sports “Regularly, more than 150 min per week” in 2024 were mostly participants earning more than twice the PSSI—2.8-fold more than persons with a lower income (Figure 1b). This observation agrees with an Australian report showing that people in lower-income households tend to report relatively lower rates of participation in sport and physical activity compared to other groups [58]. Another study performed in Flanders showed that income is positively correlated with time and money expenditure for the majority of sports activities [59]. In the EU, in 2019, nearly 55% of people in the top income group practiced sport and physical activities, as opposed to 37% of people on the lowest incomes [38]. Some sports are expensive and less accessible for people on low incomes. However, since walking, for example, does not cost anything, the level of people’s involvement in sport can probably also be explained by other factors that are not directly correlated with their financial status. These factors might include higher educational attainment, social background and position, greater awareness of the benefits of physical activity, or others [51]. It is highly plausible that these events are currently being maintained in the year of 2024, which explains this result (Figure 1b).

Quite interestingly, both sport practice and adherence to the MD increased significantly (*p* < 0.05) in 2023 when compared with 2019 and 2021 (Figure 1a and Figure 2). Other works reveal that compliance with the minimum recommendations for physical activity engagement was associated with adequate adherence to the MD [60]. However, it should be noted that the correlations are bidirectional and do not imply a cause-and-effect relationship. Therefore, determining if sport practice engagement influences adherence to the MD or the inverse is a hard task. Another Mediterranean lifestyle that showed significant differences in our study was conviviality, namely, meal companionship. Eating alone is related to an unbalanced diet, irregular eating patterns, and low subjective health, and hence is an essential public health issue [53]. Our study revealed that most of the respondents had their meals in the company of colleagues, friends, or family. Despite this, a significant decrease (*p* < 0.05) occurred in the year of 2021 relative to 2019, which may be explained by the fact that this period included the lockdown during the COVID-19 outbreak (Table 2). In some families, household members living together were concerned about the risk of transmission from eating together [61]. On the other hand, a study in Canada showed that during lockdown, many families spent more time cooking, making more meals from scratch, eating with their children more often, and involving children more often in meal preparation [57]. Regarding working conditions, the COVID-19 pandemic led people to work remotely, which reduced eating in the company of co-workers [45]. Similar phenomena were reported in students, as during the lockdown, many university students were alone during dinner time and expressed increased feelings of loneliness, leading to increased reliance on digital media and virtual commensality to socially interact with close family and friends whilst enjoying their meal [62]. The 2.5-fold greater % of single, divorced, separated, or widowed people eating alone in 2021, relative to the ones who were married or were in an analogous relationship (Figure 1b) is easily explainable when considering the information above.

## 5. Conclusions

To our knowledge, this is the first study assessing MD adherence about one year after the end of the COVID pandemic. The study highlights the dynamic nature of adherence to the Mediterranean Diet (MD) among Portuguese adults across different phases of the COVID-19 pandemic. As initially hypothesized, the adherence to the MD varied significantly among different sociodemographic groups during various phases of the pandemic, and during the confinement the exhibited trend consisted in an increase in the adhesion to this diet.

Our findings indicate an initial increase in MD adherence during the pandemic, as evidenced by the rise in MEDAS scores from 2019 to 2021, which reflects a shift towards healthier food choices and lifestyle changes during periods of confinement. However, this positive trend was not sustained over time, with a notable decline in adherence observed by 2024, following the end of the pandemic. Therefore, the initial assumption that the variations that occurred during the confinement period would be maintained over time was not validated by our results. In fact, the decline observed suggests that the temporary behavior changes adopted during the pandemic were not fully integrated into long-term habits, particularly as external factors such as economic instability emerged. The decrease in the consumption of key MD components, such as vegetables and fish, and the increase in red meat consumption underscore the challenges of maintaining healthy eating patterns post-pandemic. These findings call for targeted public health interventions to reinforce the benefits of the MD and encourage sustained healthy eating behaviors, particularly when considering the economic and social challenges that continue to influence dietary habits. Future research should explore the underlying factors contributing to these shifts and identify effective strategies for promoting long-term adherence to the MD.

In the present paper, we propose to analyze eventual changes in adherence to the MD in three Portuguese adult cohorts, covering the periods of pre-lockdown due to the COVID-19 pandemic (2019), two months after the last lockdown (2021), and about one year after the official end of the pandemic (2024), and to explore sociodemographic factors that may be associated with these changes. We aimed to determine if the adherence to the MD varied significantly among different sociodemographic groups during various phases of the pandemic and assess whether these changes were permanent or temporary. This study also underscores the evolving nature of Mediterranean lifestyle practices, including physical activity and conviviality. Our findings highlight a gradual increase in physical activity levels during the pandemic and a significant rise by 2024, suggesting a positive shift towards a more active lifestyle. However, despite this increase, a substantial proportion of the population remains insufficiently active, reflecting persistent challenges in promoting widespread engagement in regular physical activity. The observed trends in physical activity may be attributed to increased awareness of the benefits of exercise, heightened by the pandemic’s impact on mental health and well-being. Furthermore, our data suggest that socioeconomic factors, such as income and gender, continue to influence physical activity levels, with higher-income individuals and men more likely to engage in regular exercise. These findings point to the need for targeted interventions that address barriers to physical activity, particularly among low-income groups and women. Conviviality, an integral aspect of the Mediterranean lifestyle, also underwent notable changes during the pandemic. The decrease in communal eating in 2021, likely due to lockdowns and social distancing measures, highlights the social disruptions caused by the pandemic. While most participants continued to share meals with family or friends, the increase in solitary eating, especially among single, divorced, or widowed individuals, suggests a shift in social dynamics that warrants further attention.

Overall, this study provides valuable insights into the impact of the COVID-19 pandemic on Mediterranean lifestyle practices. However, a limitation of this study was the sampling method, as questionnaires were distributed via Google Forms(free version), an online platform. The ability to use and access such technology tends to be higher among individuals with higher education and/or higher income levels. Consequently, a significant percentage of participants had a university-level education; this may not accurately represent the general Portuguese population. In fact, subjects with higher education are generally more knowledgeable about the health benefits of the Mediterranean Diet [63], and higher income allows greater access to fresh, diverse food products, even during periods of economic uncertainty or food shortages, such as during the pandemics [64]. This study underscores the importance of promoting holistic approaches that integrate nutrition, physical activity and social well-being to enhance public health. Targeted interventions should focus on individuals with lower adherence to the Mediterranean Diet. Specifically, educational campaigns aimed at families, with a focus on men, should highlight the importance of reducing red meat consumption. Additionally, workplace nutrition programs should be developed for employed individuals, along with physical activity programs tailored for women and lower-income groups. Similar initiatives should be introduced in universities to raise awareness about the negative effects of excessive alcohol consumption.

From the pandemic, we may have learned several key lessons regarding health behaviors and dietary patterns. A lockdown scenario can lead to short-term improvements in dietary habits, with people adopting healthier behaviors like increased adherence to the MD and engaging in more physical activity, although sociodemographic variables, such as income and gender, had a relevant influence. Even so, the pandemic demonstrated that behavioral change is possible under the right conditions, suggesting that structured interventions, like those implemented during lockdowns, can be effective. However, it also highlights the challenge of translating these temporary changes into long-term habits, emphasizing the need for continuous public health support. Therefore, future research should explore the long-term sustainability of these lifestyle changes and develop strategies to support diverse populations in maintaining a balanced and healthful Mediterranean lifestyle.

## Figures and Tables

**Table 1:** Participants’ sociodemographic characteristics in the 2019, 2021, and 2024 cohorts.

	2019	2021	2024	*p*-Value
**N**	**500**	**375**	**207**	
Gender (%/n)				<0.001
Men	28.2/140	41.9/157	28.5/59	
Women	71.8/357	58.1/218	71.5/148	
Age (mean ± sd)	36.4 ± 13.6	40.0 ± 13.7	44.1 ± 15.6	<0.001
Age categories (%/n)				<0.001
Less than 45 years	69.8/349	63.5/238	44.0/91	
Over 45	30.2/151	36.5/137	56.0/116	
Marital status (%/n)				0.002
Married or analogous relationship	45.0/225	55.2/207	57.0/118	
Single, divorced, separated, or widowed	55.0/275	44.8/168	43.0/89	
Education level (%/n)				0.887
Without university level	27.0/135	27.5/103	25.6/51	
With university level	73.0/365	72.5/272	74.4/154	
Employment status (%/n)				0.160
Student, unemployed, housework, pensioner	29.9/149	24.1/89	26.6/55	
Employed	70.1/350	75.9/281	73.4/152	
Monthly net income (euros) (%/n)				0.682
<2 × PSSI ^(1)^	12.0/48	12.0/36	14.5/24	
>2 × PSSI	88.0/353	88.0/265	85.5/142	

^(1)^ PSSI is the Portuguese Social Support Index in 2019 to 2021 (EUR 438.81) and in 2024 (EUR 509.26). N, sample size; n, number of participants; sd, standard deviation. To assess differences among periods, Mann–Whitney U tests were used for ordinal and scale variables while Chi square tests were used for nominal variables. Differences were considered significant when *p*-values < 0.05.

**Table 2:** Participants’ lifestyle characteristics in the 2019, 2021, and 2024 cohorts.

	2019	2021	2024	*p*-Values		
Sports practice (%/N)						
Never or occasionally	59.4/225	55.2/144	40.9/56	Year	2021	2024
Regularly, less than 150 min per week	18.2/69	20.7/54	30.7/42	2019	0.414	**0.001**
Regularly, more than 150 min per week	22.4/85	24.1/63	28.5/39	2021	-	**0.012**
Sleep habits (%/N)				Year	2021	2024
Less than seven hours per night	59.4/297	58.7/220	56.5/117	2019	0.469	0.200
Eight or more hours per night	40.6/203	41.3/155	43.5/90	2021	-	0.515
Time spent in nature (%/N)						
Never or occasionally	33.4/167	36.0/135	43.0/89	Year	2021	2024
Sometimes	35.8/179	32.8/123	30.9/64	2019	0.708	**0.030**
Frequently or almost all the time	30.8/154	31.2/117	26.1/54	2021	-	0.075
Time spent with family (%/N)						
Never or occasionally	14.0/70	15.2/57	10.1/21	Year	2021	2024
Sometimes	21.8/109	24.8/93	26.6/55	2019	0.208	0.892
Frequently or almost all the time	64.2/321	60.0/225	63.3/131	2021	-	0.387
Time spent with friends (%/N)						
Never or occasionally	24.8/124	54.7/205	38.2/79	Year	2021	2024
Sometimes	39.8/199	30.1/113	32.4/67	2019	**0.000**	**0.029**
Frequently or almost all the time	35.4/177	15.2/57	29.5/61	2021	-	**0.000**
Daily meal companionship (%/N)				Year	2021	2024
Alone	17.4/87	23.2/87	18.4/38	2019	**0.005**	0.259
In the company of colleagues, friends, or family	82.6/413	76.8/288	81.6/168	2021	-	0.262

Data are expressed as frequencies (%) and sample size (N). To access differences among periods, Quade non-parametric ANCOVA tests were used, fixing confounders (the sociodemographic variables sex, age, and marital status) as covariates. Differences were considered significant when *p*-values < 0.05 (in bold).

**Table 3:** Participants’ Mediterranean Diet adherence expressed by the MEDAS score in the 2019, 2021, and 2024 cohorts, and the categories of adherence: weak, moderate-to-fair, and good or very good.

MEDAS	2019	2021	2024	*p* Value		
				Year	2021	2024
(mean ± sd/N)	6.2 ± 0.7/500	7.7 ± 0.1/375	7.2 ± 0.1/207	2019	**0.000**	**0.000**
				2021	-	**0.010**
(%/N)						
Weak (≤5)	31.6/158	13.1/49	15.9/33	Year	2021	2024
Moderate to fair (6 to 9)	67.4/337	68.8/258	75.5/157	2019	**0.000**	**0.000**
Good or very good (≥10)	1.0/5	18.1/68	8.2/17	2021	-	**0.004**

Data are expressed as frequencies (%) and sample size (N). To access differences among periods, Quade non-parametric ANCOVA tests were used, fixing confounders (sex, age class, and marital status) as covariates. Differences were considered significant when *p*-values < 0.05 (in bold).

**Table 4:** Frequency of consumption of the 14 individual food items of the MEDAS score, reported in the 2019, 2021, and 2024 cohorts.

	2019	2021	2024	*p* Value		
Use of olive oil to cook (%/N)				Year	2021	2024
No	3.4/17	2.4/9	1.9/4	2019	0.303	0.770
Yes *	96.6/483	97.6/366	98.1/203	2021	-	0.593
Spoons of olive oil per day (%/N)						
One or less	36.8/184	32.3/121	41.5/86	Year	2021	2024
Two or three	53.8/269	58.9/221	49.8/103	2019	0.265	0.161
Four or more *	9.4/47	8.8/33	8.7/18	2021	-	**0.027**
Vegetable portions per day (%/N)						
Less than one	11.2/56	6.9/26	15.5/32			
One	35.2/176	30.4/114	44.0/91	Year	2021	2024
Two *	38.4/192	45.6/171	28.5/59	2019	**0.005**	**0.000**
Three or more	15.2/76	17.1/64	12.1/25	2021	-	**0.000**
Fruit portions per day (%/N)						
Less than one	13.8/69	8.5/32	10.6/22			
One	23.6/118	24.8/93	26.6/55	Year	2021	2024
Two	39.0/195	34.9/131	40.6/84	2019	**0.008**	0.284
Three or more *	23.6/118	31.7/119	22.2/46	2021	-	**0.002**
Portions of red meat and derivates per week (%/N)						
One or less	35.6/178	40.0/150	31.4/65			
Two to four	44.0/220	41.1/154	44.9/93	Year	2021	2024
Five to six *	16.0/80	13.3/50	18.4/38	2019	0.066	0.202
Seven or more	4.4/22	5.6/21	5.3/11	2021	-	**0.008**
Fat portions per day (margarine, butter, cream) (%/N)						
Less than one *	68.0/340	70.1/263	63.8/132	Year	2021	2024
One	22.6/113	23.2/87	28.5/59	2019	0.314	0.548
More than one	9.4/47	6.7/25	7.7/16	2021	-	0.171
Sweet beverages per day (%/N)						
Less than one *	82.6/413	91.2/342	84.5/175	Year	2021	2024
One	11.8/59	6.1/23	12.6/26	2019	**0.000**	0.815
More than one	5.6/28	2.7/10	2.9/6	2021	-	**0.003**
Wine per week (%/N)						
One or less (occasionally)	75.6/378	77.9/292	75.8/157			
Two to six (sometimes but not daily)	19.6/98	18.4/69	19.8/41	Year	2021	2024
Seven to fourteen (one or two glasses per day) *	4.2/21	3.5/13	3.4/7	2019	**0.017**	0.121
More than fourteen (more than two glasses per day)	0.6/3	0.3/1	1.0/2	2021	-	0.680
Legumes per week (%/N)						
Less than one	17.6/88	22.9/86	22.2/46			
One	30.8/154	28.5/107	25.1/52	Year	2021	2024
Two	28.4/142	27.5/103	35.3/73	2019	0.115	0.257
Three or more *	23.2/116	21.1/79	17.4/36	2021	-	0.872
Fish per week (%/N)						
Less than one	11.8/59	7.7/29	13.0/27			
One	17.8/89	13.9/52	19.3/40	Year	2021	2024
Two	35.8/179	38.7/145	36.2/75	2019	**0.013**	0.106
Three or more *	34.6/173	39.7/149	31.4/65	2021	-	**0.000**
Sweets per week (%/N)						
Less than one	26.6/133	27.7/104	23.2/48			
One	27.8/139	24.3/91	19.8/41			
Two *	18.4/92	24.0/90	24.6/51	Year	2021	2024
Three	10.6/53	14.7/55	16.4/34	2019	0.315	0.058
Four or more	16.6/83	9.3/35	15.9/33	2021	-	**0.009**
Nuts per week (%/N)						
Less than one	34.6/173	31.7/119	32.9/68			
One	18.0/90	17.3/65	27.1/56	Year	2021	2024
Two	18.0/90	22.1/83	21.7/45	2019	0.584	**0.008**
Three or more *	29.4/147	28.8/108	18.34/38	2021	-	**0.003**
Red meat restriction (%/N)				Year	2021	2024
No	23.4/117	22.9/86	21.7/45	2019	0.258	0.317
Yes	76.6/383	77.1/289	78.3/162	2021	-	0.911
Dishes cooked with tomatoes or tomato sauce, onion and (or) garlic, and olive oil per week (%/N)						
Less than one	12.2/61	13.1/49	12.1/25	Year	2021	2024
One	20.2/1010	19.7/74	21.3/44	2019	0.665	0.720
Two or more *	67.6/338	67.2/252	66.7/138	2021	-	1.000

The threshold for the recommended frequencies is indicated with *. Differences among 2019, 2021, and 2024 cohorts were assessed by Quade non-parametric ANCOVA tests, fixing confounders (sex, age, and marital status) as covariates. Differences were considered significant when *p*-values < 0.05 (in bold).

## Data Availability

The original contributions presented in the study are included in the article and Supplementary Materials, further inquiries can be directed to the corresponding author.

## Supplementary Materials

The following supporting information can be downloaded at: https://www.mdpi.com/article/10.3390/nu16193372/s1, Figure S1: Lifestyle parameters for which significant differences (*p* < 0.05) among the years 2019, 2021, and 2024 were observed. (a) Sport practice, (b) time spent in nature, (c) time spent with friends, and (d) daily meal companionship; Figure S2: Diet parameters for which significant differences (*p* < 0.05) among the years of 2019, 2021, and 2024 were observed. (a) MEDAS score; (b) red meat portions per day; (c) sweets per week; (d) nut portions per week; (e) spoons of olive oil per day; (f) vegetable portions per day; (g) fruit portions per day; (h) sweet beverages per day; (i) wine per week; (j) fish portions per week.
