# Supplementary material for: Fluctuations in Mediterranean Diet Adherence Pre- and Post-Pandemic: A Study of Portuguese Cohorts 2019–2024
Source: Nutrients. 2024 Oct 3;16(19):3372. doi: 10.3390/nu16193372 (PMC11478962; doi:10.3390/nu16193372)
Supplement: Supplementary file 1 [file nutrients-16-03372-s001.zip › nutrients-3244543-supplementary.pdf]

Supplementary materials

a)

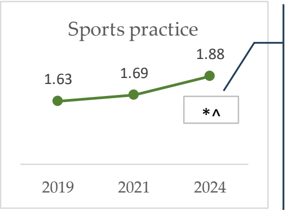

Regularlly, more than 150 minutes per week (%:%/ratio)	
**Men**/Women	27.7:16.9/3.0
**More**/Less, **than 45 years**	20.7:16.5/1.3
**Alone**/Relationship	22.4:16.1/1.4
**With**/Without **university**	20.1:15.7/1.3
**Not working**/Working	19.7:16.4/1.2
</> **2xPSSI**	19.7:8.3/2.8

b)

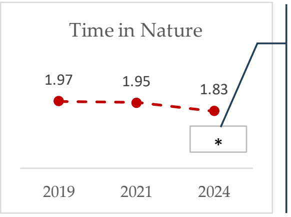

Never or occasionally (%:%/ratio)	
**Women**/Men	43.9:40.7/1.1
**More**/Less, **than 45 years**	43.1:42.9/1.0
**Alone**/Relationship	48.3:39.0/1.2
**Without**/With **university**	49.0:41.6/1.2
**Not working**/Working	44.7:38.2/1.2
</> **2xPSSI**	45.8:44.4/1.0

c)

Never or occasionally (%:%/ratio)	
**Women**/Men	56.0:52.9/1.1
**More**/Less, **than 45 years**	57.7:52.9/1.1
**Relationship**/Alone	62.8:44.6/1.4
**Without**/With **university**	57.3:53.7/1.1
**Not working**/Working	56.9:47.2/1.2
>/< **2xPSSI**	58.9:52.8/1.1

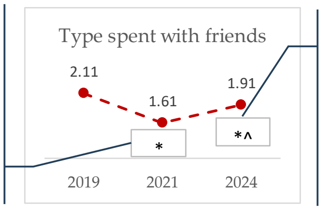

Never or occasionally (%:%/ratio)	
**Men**/Women	42.8:36.5/1.2
**More**/Less, **than 45 years**	41.4:34.1/1.2
**Relationship**/Alone	44.9:29.2/1.5
**Without**/With **university**	45.1:36.4/1.2
**Not working**/Working	42.1:27.3/1.5
>/< **2xPSSI**	38.0:29.2/1.3

d)

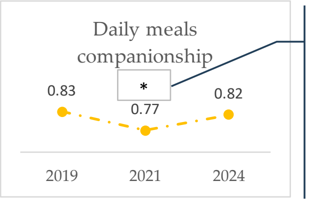

Alone (%:%/ratio)	
**Men**/Women	24.2:22.5/1.1
**Less**/More, **than 45 years**	24.0:21.9/1.1
**Alone**/Relationship	34.5:14.0/2.5
**With**/Without **university**	22.8:22.3/1.0
**Not working**/Working	23.8:21.3/1.1
>/< **2xPSSI**	20.6:13.5/1.5

Figure S1. Lifestyle parameters for which significant diferences (p<0.05) among the years 2019, 2021 and 2024 were observed and sociodemographic profiles of participants. a) sport practice, b) time spent in nature, c) Time spent with friends, and d) Daily meals companionship. Values of lifestyle parametres are expressed as mean score values (Sport Practice: “Never or occasionally” = 1, “Regularly, less than 150 min per week” = 2 and “Regularly, more than 150 min per week” = 3; Time spent in Nature, with Family or with Friends, “Never or occasionally” = 1, “Some-times” = 2 and “Frequently or almost all the time”=3; Daily meals companionship, “Alone” = 0 and “In the company of colleagues, friends or family”=1). To acess diferences among periods, Quade Non-parametric ANCOVA tests were used, fixing confounders (the sociodemographic variables sex, age and marital status) as covariates. Differences were considered significant when p-values < 0.05: * is diferent from 2019 and ^ is diferent form 2021. The tables show the ratio of relative %s of sociodemographic classes of the participants in the upper frequency category for variables that increased, and of the participants in the lower frequency category for variables that decreased.

a)

7.67 ± 2.0 (moderate/good adherence) (%:%/ratio)	
**Women**/Men	72.5:63.7/1.1
**Less**/More, **than 45 years**	68.9:68.6/1.0
**Relationship**/Alone	70.0:67.3/1.0
**Without**/**With university**	76.7:65.8/1.2
**Working**/Not working	71.9:67.6/1.1
</> **2xPSSI**	69.4:68.3/1.0

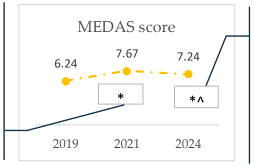

7.24 ± 1.7 (moderate adherence) (%:%/ratio)	
**Women**/Men	61.5:57.6/1.1
**Less**/More, **than 45 years**	68.1:54.3/1.3
**Alone**/Relationship	66.3:55.9/1.2
**With**/**Without university**	62.3:56.9/1.1
**Working**/Not working	74.5:56.5/1.3
>/< **2xPSSI**	55.9:23.6/2.4

b)

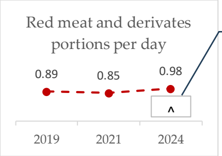

Seven or more portions per week (%:%/ratio)	
**Men**/Women	10.2:3.4/3.0
**Less**/More, **than 45 years**	5.5:5.2/1.1
**Relationship**/Alone	7.6:2.3/3.3
**Without**/**With university**	7.8:4.6/1.7
**Not working**/Working	6.6:1.8/3.7
>/< **2xPSSI**	5.6:4.2/1.3

c)

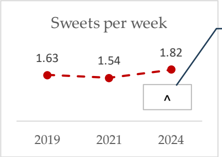

More than twice per week (%:%/ratio)	
**Men**/Women	40.7:29.1/1.4
**More**/Less, **than 45 years**	35.4:28.6/1.2
**Relationship**/Alone	34.8:29.2/1.2
**Without**/**With university**	47.1:27.9/1.7
**Working**/Not working	41.8:28.9/1.4
>/< **2xPSSI**	35.0:25.0/1.4

d)

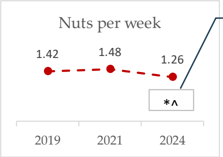

Less than three times per week (%:%/ratio)	
**Men**/Women	91.5:77.7/1.2
**Less**/More, **than 45 years**	91.2:74.1/1.2
**Alone**/Relationship	87.6:77.1/1.1
**With**/**Without university**	83.1:80.4/1.0
**Working**/Not working	90.9:78.3/1.2
</> **2xPSSI**	83.3:80.3/1.0

e)

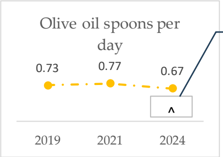

Less than four spoons per day (%:%/ratio)	
**Women**/Men	92.6:88.1/1.1
**Less**/More, **than 45 years**	93.4:89.6/1.0
**Alone**/Relationship	96.6:87.3/1.1
**Without**/**With university**	98.0:90.3/1.1
**Working**/Not working	96.4:89.5/1.1
</> **2xPSSI**	95.8:92.6/1.0

f)

More than one poortion per day (%:%/ratio)	
**Women**/Men	65.6:58.6/1.1
**More**/Less, **than 45 years**	67.2:60.1/1.1
**Relationship**/Alone	62.8:62.5/1.0
**With**/**Without university**	63.2:61.2/1.0
**Not working**/Working	64.8:53.9/1.2
>/< **2xPSSI**	62.6:58.3/1.1

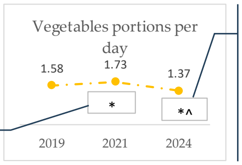

Less than two portions per day (%:%/ratio)	
**Women**/Men	69.5:55.4/1.3
**Less**/More, **than 45 years**	71.4:50.0/1.4
**Alone**/Relationship	66.3:54.2/1.2
**Without**/**With university**	60.8:59.7/1.0
**Working**/Not working	65.5:57.2/1.1
</> **2xPSSI**	66.7:56.3/1.2

g)

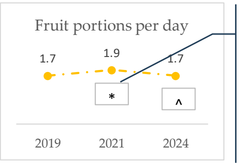

Three or more portions per day (%:%/ratio)	
**Men**/Women	32.5:31.2/1.0
**More**/less, **than 45 years**	40.6:26.5/1.5
**Alone**/Relationship	32.7:30.9/1.1
**With**/**Without university**	32.0:31.1/1.0
**Not working**/working	35.5:23.6/1.5
</> **2xPSSI**	36.1:29.8/1.2

h)

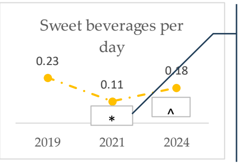

Less than one per day (%:%/ratio)	
**Women**/Men	92.7:89.2/1.0
**More**/less, **than 45 years**	93.4:89.9/1.0
**Relationship**/Alone	91.3:91.1/1.0
**Withou**/**With university**	91.3:91.2/1.0
**Not working**/working	91.1:87.6/1.0
>/< **2xPSSI**	93.2:80.6/1.2

i)

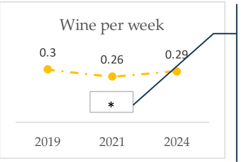

One or two glasses per day (%:%/ratio)	
**Men**/Women	92.7:89.2/1.0
**More**/less, **than 45 years**	7.3:1.3/5.6
**Relationship**/Alone	3.9:3.0/1.3
**With**/**Without university**	3.7:2.5/1.5
**Not working**/working	3.6:2.5/1.4
>/< **2xPSSI**	4.3:1.1/3.9

j)

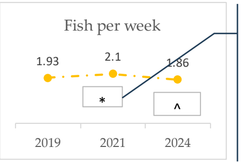

Three or more per week (%:%/ratio)	
**Women**/Men	41.3:37.6/1.1
**More**/less, **than 45 years**	45.3:36.6/1.2
**Relationship**/Alone	43.0:35.7/1.2
**Without**/**With university**	47.6:36.8/1.3
**Not working**/working	42.7:30.3/1.4
</> **2xPSSI**	41.7:40.8/1.0

Figure S2. Diet parameters for which significant diferences ($p<0.05$) among the years of 2019, 2021 or 2024 were observed and sociodemographic profiles of participants. a) MEDAS Score; b) red meat portions per week; c) sweets per week; d) nuts portions per week; e) olive oil spoons per day; f) vegetable portions per day; g) fruit portions per day; h) sweet beverages per day; i) wine per week; j) fish portions per week. MEDAS score was calculated as reported previously [11] (0 = minimum, 14 = maximum). Individual food items are expressed as mean frequency score values (portions of red meat and derivate per week, "One or less" =0, "Two or four = 1; "Five to six" =2 and "Seven or more =3"; sweets per week, "Less than one"=0, "One"=1; "Two" = 2; "Three =3" and "Four or more"=4; portions of nuts and fish per week, portions of vegetables and fruit per day, "Less than one" = 0; "One" = 1; "Two" = 2 and "Three or more"=3; olive oil spoons per day, "One or less"=0, "Two or three" = 1 and "Four or more"=2; sweet beverages per day, "Less than one"=0, "One"=1 and "More than one" =2; wine, "Occasionally" = 0, "Sometimes but not daily" = 1, "One or two glasses per day"=2, "More than two glasses per day" = 3. To acess diferences among periods, Quade Non-parametric ANCOVA tests were used, fixing confounders (the sociodemographic variables sex, age and marital status) as covariates. Differences were considered significant when p-values < 0.05: * is diferent from PreC and ^ is diferent form 1stPosC. The tables shows the sociodemographic characteristics of the participants falling within the MD recommendations, in case of a trend towards recommendation, or participants not meeting the MD recommendations, in case of a trend away from recommendations.
